# Modulation of Indian monsoon by water vapor and cloud feedback over the past 22,000 years

**DOI:** 10.1038/s41467-019-13754-6

**Published:** 2019-12-13

**Authors:** Chetankumar Jalihal, Jayaraman Srinivasan, Arindam Chakraborty

**Affiliations:** 1 Centre for Atmospheric and Oceanic Sciences, Indian Institute of Science, Bangalore, 560012 India; 2 Divecha Centre for Climate Change, Indian Institute of Science, Bangalore, 560012 India

**Keywords:** Atmospheric dynamics, Climate and Earth system modelling, Palaeoclimate

## Abstract

To predict how monsoons will evolve in the 21st century, we need to understand how they have changed in the past. In paleoclimate literature, the major focus has been on the role of solar forcing on monsoons but not on the amplification by feedbacks internal to the climate system. Here we have used the results from a transient climate simulation to show that feedbacks amplify the effect of change in insolation on the Indian summer monsoon. We show that during the deglacial (22 ka to 10 ka) monsoons were predominantly influenced by rising water vapor due to increasing sea surface temperature, whereas in the Holocene (10 ka to 0 ka) cloud feedback was more important. These results are consistent with another transient simulation, thus increasing confidence despite potential model biases. We have demonstrated that insolation drives monsoon through different pathways during cold and warm periods, thereby highlighting the changing role of internal factors.

## Abstract (web summary)

Past Indian summer monsoon (ISM) changes are not well understood. The application of an energetic framework to a transient model simulation shows that ISM influences have changed in the past, with rising water vapor more important during deglaciation, whereas cloud feedbacks dominated during the Holocene.

## Introduction

The changes in the Earth–Sun geometry affect the monsoon by altering the solar energy available for sustaining the monsoons^1–4^. Some studies have shown that the southern hemisphere insolation can also affect the Indian monsoon through latent heat transport^5–7^, highlighting the role of feedbacks from the southern hemisphere. The different paths through which solar insolation can influence the monsoon have been outlined^8^, but so far a detailed mechanism has not been proposed. Moreover, a definitive understanding of the role of forcing and feedback is missing. Therefore, a diagnostic model is useful to identify the important parameters that influence the monsoon. One such model is based on the energy and moisture budget of monsoons and is known as the energetics framework.

The energetics framework considers monsoon as an energetically direct circulation system that exports excess energy to regions with an energy deficiency^9^. The traditional view of monsoons based on the land–sea thermal contrast cannot explain why precipitation peaks in July even though the highest land temperatures are in May^10^. After the onset of monsoons, the land cools due to increased cloud cover and higher evaporation^10^. Hence, the energetics approach is superior to the land–sea thermal contrast theory^9^. A version of the energetics framework has been used extensively to understand the meridional movement of the zonal mean intertropical convergence zone (ITCZ)^11–15^. Asymmetry in the interhemispheric temperature drives these meridional shifts. While the changes in the Earth–Sun geometry affect the interhemispheric temperature distribution, the zonal asymmetries in net energy into the atmosphere have a greater impact on monsoon rainfall^16,17^. These asymmetries arise due to differences in the net surface energy fluxes over land and oceans. The zonal variations in ITCZ can also be studied with this framework^18,19^. The difference between the energetics of the zonally averaged ITCZ, and regional monsoons is aptly summarized by the phrase—global energetics versus local physics^9^. Using this framework in idealized time-slice experiments, it was shown that the Indian monsoon is directly driven by insolation^16^. On the other hand, it is known that greenhouse gases and ice sheets affect monsoons by modulating water vapor^20–22^. Therefore, the following questions arise: how do simultaneous variations in greenhouse gases and ice sheets influence the monsoon response to solar insolation? What role does water vapor play under such circumstances?

A study of the evolution of monsoon over the last 22,000 years is ideal for addressing these questions. Earth’s climate underwent dramatic changes over the last 22,000 years. The ice age was at its peak around 21,000 years ago. Greenhouse gas (GHG) concentrations were much lower^23^ and ice sheets extended up to midlatitudes^24–26^. During the deglacial the GHG concentrations increased, ice sheets receded, and both of them started influencing the monsoons^27–29^. The Holocene was relatively stable with changes in solar insolation being the dominant forcing (see Supplementary Note 1 for further details).

In this study, we have discerned the role of feedbacks in modulating the effect of solar insolation on the Indian summer monsoon rainfall (ISMR). Our results reveal that water vapor amplifies the solar forcing during cold climates, whereas cloud feedback is dominant in warm climates. Simultaneous forcings from greenhouse gases and ice sheets during the deglacial further intensify the effect of water vapor. Thus, ISMR has different sensitivities to solar forcing during the deglacial and the Holocene.

## Results

### The deglacial versus the holocene

Here we have used a transient simulation, known as TraCE-21k^30,31^ to study the Indian monsoon over the last 22,000 years. It is a simulation with transient forcings (greenhouse gas concentrations, ice-sheet extent, orbital parameters, and meltwater fluxes) in a fully coupled ocean–atmosphere global circulation model CCSM3 over the last 22,000 years. The variations in ice sheets and GHG are related to internal climate feedbacks in response to orbital forcing^32–36^. In the TraCE-21k ice sheets and GHG are imposed, hence, strictly not feedback on orbital forcing. Therefore, we treat them as a separate forcing on monsoon. Our approach is concerned more with the moisture convergence over well-defined monsoon regions via the vertically integrated moist static energy budget as used in previous studies (see ref. ^37^ and references within).

**Fig. 1:**
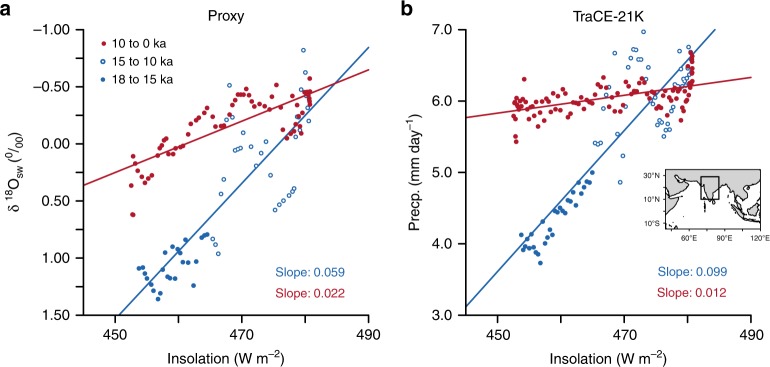
The sensitivity of the Indian summer monsoon to insolation. **a** The scatter of ${\delta }^{18}{{\rm{O}}}_{{\rm{sw}}}$ from the core KL-126 from the Bay of Bengal^38^ and summer insolation (Jun–Jul–Aug) over India (10${}^{\circ }$–29${}^{\circ }$N and 70${}^{\circ }$–85${}^{\circ }$E). The ${\delta }^{18}{{\rm{O}}}_{{\rm{sw}}}$ represents salinity in the northern Bay of Bengal (from where the sediment core is taken), and is influenced by precipitation over the Indian subcontinent. Thus, ${\delta }^{18}{{\rm{O}}}_{{\rm{sw}}}$ is a proxy for the Indian summer monsoon. **b** The scatter of Indian summer monsoon rainfall (Jun–Jul–Aug) versus insolation over India from the TraCE-21k dataset. The blue and red filled circles denote the time periods (18–15 ka) and (10–0 ka), respectively. Open blue circles indicate the time period between (15 and 10 ka). This period experienced large centennial to millennial-scale excursions. Every circle represents an average over a century. The region chosen for this study is outlined with a black box within the inset (only land grids were considered). The blue and the red lines are the least-square fit for the periods (18–10 ka) and (10–0 ka), respectively.

The ISMR is more sensitive to insolation during the deglacial period than during the Holocene (Fig. 1a), as indicated by a proxy. This proxy is based on the sea surface salinity (SSS) in the Bay of Bengal, as inferred from the ${\delta }^{18}$O from the core KL-126^38^. The rainfall over the Indian subcontinent influences the SSS over the Bay of Bengal through river runoffs. Thus, variations in SSS indicate variations in the strength of ISMR. The ${\delta }^{18}$O is, however, also a function of sea surface temperature (SST) and ice volume. Hence, the effect of SST and ice volume is removed to obtain ${\delta }^{18}{{\rm{O}}}_{{\rm{sw}}}$ (see the “Methods” section). The larger negative values of ${\delta }^{18}{{\rm{O}}}_{{\rm{sw}}}$ correspond to stronger ISMR. The TraCE-21k also exhibits a difference in the sensitivity of the deglacial monsoon and the Holocene monsoon to solar forcing. TraCE-21k is known to have a dry bias in the African monsoon and also underestimates its northward expansion during the Holocene^39^. The long-term trends in the African and East Asian monsoons are, however, simulated quite well^27,40^. TraCE-21k also has a good representation of the ISMR (see Supplementary Figs. 1, 2, and 3) with a bias ranging from −16 to −21% during the present-day climate, depending on which an observational dataset is used (see Methods for more details). TraCE-21k is well within the spread of the CMIP5/PMIP3 models (see Supplementary Figs. 1 and 3). Since we are addressing the different sensitivity of the ISMR between the deglacial and the Holocene, this bias does not affect our results (see Supplementary Fig. 3). To identify the mechanism responsible for the different sensitivities of ISMR during these two periods, we have used a diagnostic model based on the conservation of energy and moisture over the monsoon region^41^. It has been shown that (see Methods for the derivation)

1 $$P-E=\frac{{Q}_{{\mathrm{net}}}}{{\mathrm{GMS}}}$$

**Fig. 2:**
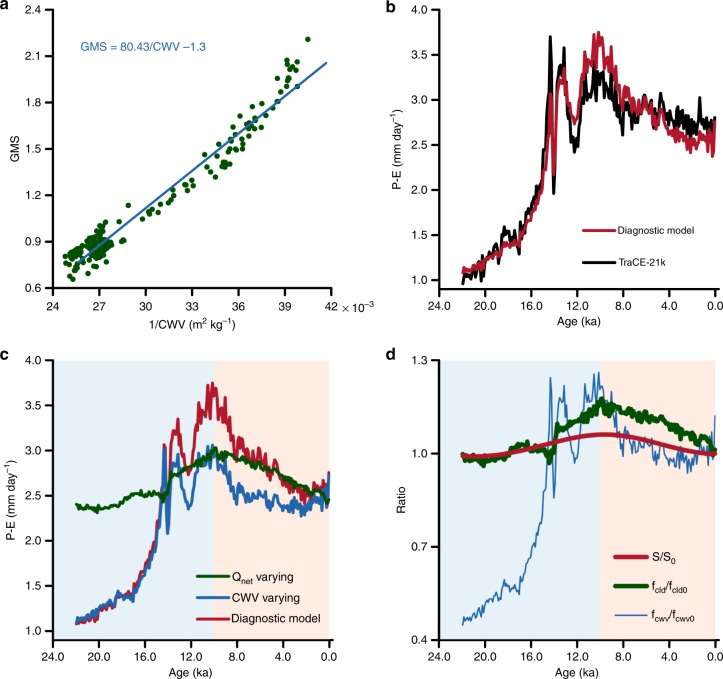
The Indian summer monsoon as a function of net energy and water vapor. **a** The scatter between gross moist stability (GMS) and total column water vapor (CWV) over India. **b** The time series of moisture convergence ($P-E$) over India from the TraCE-21k (black) and the diagnostic model (red). **c** The time series of $P-E$ from the diagnostic model over India under three conditions, namely, only net downward radiative flux at the top of the atmosphere (${Q}_{{\rm{net}}}$) varies, and CWV is held constant at its preindustrial value (in green), only CWV varies, whereas ${Q}_{{\rm{net}}}$ is fixed at its preindustrial value (in blue), and, finally both CWV and ${Q}_{{\rm{net}}}$ vary (in red). **d** The time series of solar insolation ($S$) in red, cloud radiative feedbacks (*f*_cld_) in blue, and the effect of water vapor (${f}_{{\rm{cwv}}}$) in green, normalized with respect to their preindustrial values. The preindustrial climate is obtained by taking the average over the period 1750–1850 AD. The background colors indicate the two periods classified based on the dominance of water vapor (shown in blue) or ${Q}_{{\rm{net}}}$ (shown in red).

This model indicates that the moisture convergence (i.e., rainfall–evaporation; $P-E$; units—mm day${}^{-1}$) is directly proportional to the net downward radiative flux at the top of the atmosphere (${Q}_{{\rm{net}}}$; units—mm day${}^{-1}$; 28.9 W m${}^{-2}$ = 1 mm day${}^{-1}$) and inversely proportional to the gross moist stability (GMS). ${Q}_{{\rm{net}}}$ includes some influence from cloud cover, greenhouse gases including water vapor, and changes in albedo (e.g., due to changes in vegetation). ${Q}_{{\rm{net}}}$ can be written as a product of solar insolation and cloud feedbacks *f*_cld_ (see Methods). The GMS over a region, is a measure of net lateral outflow of moist static energy per unit moisture converged into that region^42^. The GMS amplifies or dampens the impact of solar insolation. We show that it is only a function of total column water vapor (CWV; units—kg m${}^{-2}$) (Fig. 2a). This relation between GMS and CWV for the centennially averaged Jun–Jul–Aug (JJA) mean climate is similar but not identical to the result obtained for the seasonal cycle by a previous study^43^.

For the range of values of GMS and 1/CWV in the TraCE-21k, it is sufficient to use a linear fit between the two quantities. For an extended range of values, the relation is nonlinear with no real roots (see Supplementary Fig. 9). Therefore, the diagnostic model is stable for all real values of CWV. All the processes resulting from an insolation forcing that affects monsoon are encapsulated in CWV and ${Q}_{{\rm{net}}}$. The solar insolation can affect monsoons by changing the local ${Q}_{{\rm{net}}}$ (a direct effect) or by changing CWV, for example, by driving changes in sea surface temperature (an indirect effect). Water vapor plays three important roles in the monsoon. For a given mass convergence, an increase in moisture availability will result in more rainfall. Higher water vapor promotes vertical convection by affecting the static stability of the atmosphere^44–46^. Finally, water vapor is a powerful greenhouse gas, and hence, leads to strong positive feedback on surface temperature. Even though this feedback will appear in ${Q}_{{\rm{net}}}$ through OLR, ${Q}_{{\rm{net}}}$ follows the general structure of insolation and cloud feedbacks (see Supplementary Fig. 4). Hence, the effect of changes in water vapor essentially materializes through GMS.

The variations in moisture convergence (i.e., $P-E$) obtained from Eq. (11), are similar to those in the TraCE-21k simulation during the deglaciation period as well as the Holocene (Fig. 2b). In the simulation, over the deglacial period, an increase in CWV leads to a rise in $P-E$, but across the Holocene, the decline in $P-E$ is consistent with the decrease in ${Q}_{{\rm{net}}}$ (Fig. 2c). CWV rises rapidly during the deglacial before leveling off at the onset of the Holocene (see Supplementary Fig. 10). The variations in CWV, and hence in GMS, are strongly associated with those in $P-E$ during the deglacial period. We chose the crossover point of the blue and green curve at 10 ka in Fig. 2c, as the transition point from CWV-dominant regime to the ${Q}_{{\rm{net}}}$ dominant regime.

2 $$P-E=S\left(\frac{{Q}_{{\rm{net}}}}{S}\right)\left(\frac{1}{{\mathrm{GMS}}}\right)=S\cdot {f}_{{\mathrm{cld}}}\cdot {f}_{{\rm{cwv}}}$$

Equation (1) can be rewritten as a product of solar forcing and feedback from clouds (${Q}_{{\rm{net}}}/S$) and an indirect effect through water vapor ($1/{\mathrm{GMS}}$). Figure 2d elucidates the role of the indirect effect during the deglacial period. The cloud feedbacks were relatively small. Across most of the Holocene, cloud feedbacks played a major role in amplifying the solar forcing. The relative role of the forcing, the cloud feedback, and indirect effect can be evaluated quantitatively (see Supplementary Table 1). These results are valid in another transient simulation: LOVECLIM DG_ns^47^ (see Supplementary Methods and Supplementary Fig. 13), thus providing further confidence in our results.

### Effect of individual forcings

We consider simulations where only one forcing was allowed to vary, to verify the unique relation between GMS and CWV. This will also help discern the cause of large changes in CWV during the deglacial. These simulations with individual forcing, namely, ORB (orbital only), GHG (greenhouse gases only), and ICE (ice sheets only) have fixed boundary conditions of 22 ka. For these simulations, we decompose the changes in $P-E$ into contributions from ${Q}_{{\rm{net}}}$ and GMS^16^ (see Methods). All the simulations (TraCE-21k, ORB, GHG, and ICE) exhibit an inverse relation between GMS and CWV (see Supplementary Fig. 9), underscoring the versatility of this diagnostic method. The rapid increase in CWV during the deglacial is mainly due to the increase in SST driven by rising greenhouse gases (see Supplementary Fig. 10). Since there is a small increase in greenhouse gases during the Holocene, the changes in CWV are also low.

**Fig. 3:**
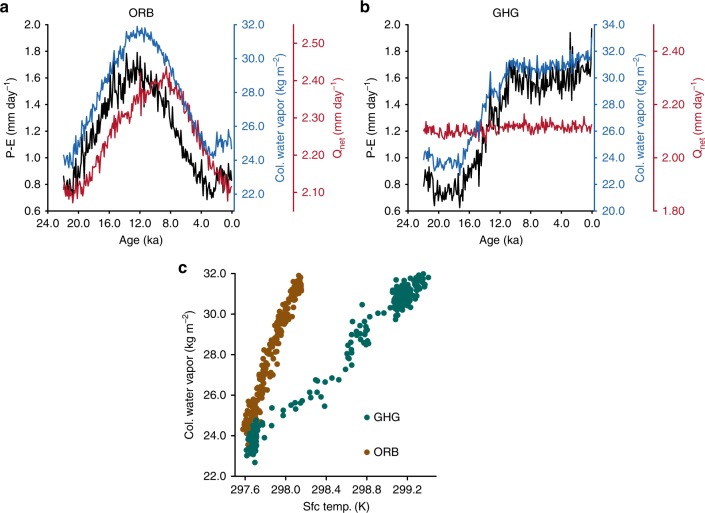
Monsoon response to individual forcings. The time series of $P-E$ (black), total column water vapor, CWV (blue) and net downward energy flux at the top of the atmosphere, ${Q}_{{\rm{net}}}$ (red) for the **a** orbit-only simulation (ORB), and **b** greenhouse gas-only simulation (GHG). **c** A scatter plot between surface temperature over the region (0${}^{\circ }$–29${}^{\circ }$N; 50${}^{\circ }$–70${}^{\circ }$E) and CWV over India (10${}^{\circ }$–29${}^{\circ }$N; 70${}^{\circ }$–85${}^{\circ }$E) for the ORB (brown), and GHG (green) simulations.

Since ORB and GHG exhibit the largest changes in CWV, we consider these simulations for a detailed analysis (see Supplementary Fig. 6). The $P-E$ over India in the ORB decreased by 42% over a period of 10,000 years (from 10 to 0 ka), even though *Q*_net_ reduced by only 12% (2.4 mm day${}^{-1}$ at 10 ka to 2.1 mm day${}^{-1}$ at 0 ka) over the same period (Fig. 3a). Besides, the peak in *Q*_net_ and $P-E$ does not coincide. Thus, changes in *Q*_net_ alone cannot explain the variations in $P-E$. Based on the analysis detailed in the Methods section, we find that CWV contributes to about 72% of this change in $P-E$. This is a surprising result, which suggests that the indirect effect of insolation on monsoons is prevalent during colder glacial conditions. Comparing ORB and TraCE-21k over the period from 10 to 0 ka (where orbital forcing is the dominant forcing in TraCE-21k and the only forcing in ORB), brings out the differences in the mechanism between the two simulations. ORB continues to have an ice-sheet extent and CO${}_{2}$ concentrations that are the same as those at LGM, whereas TraCE-21k has near-modern ice sheets and CO${}_{2}$ concentrations. Over the Holocene, insolation is the dominant parameter driving $P-E$ in TraCE-21k, whereas in the ORB simulation CWV contributes substantially to variations in $P-E$. This implies that the pathway through which insolation affects monsoons depends on the background climate state. This is due to the changing sensitivity of GMS to CWV with the mean climate (see Supplementary Fig. 9). GMS is less sensitive to CWV in warmer climates (higher water vapor) and more sensitive to CWV in colder climates (lower water vapor). In the ORB simulation, CWV and $P-E$ evolve in step with each other. Since CWV is proportional to SST (Fig. 3c), and SST lags insolation by about a month, the May–Jun–Jul insolation and CWV correspond to each other (Supplementary Fig. 7a).

In GHG-only simulation, greenhouse gases alone were allowed to vary. The concentration of CO${}_{2}$ went up from about 180–260 ppm over a span of 8000 years. In the context of anthropogenic global warming, it has been shown that monsoon precipitation increases, even though the monsoon circulation itself might weaken^48,49^. This is related to the increase in CWV due to higher SST^20,21^. Our diagnostic model also shows that CWV is the dominant driver of monsoon in GHG (since solar insolation is constant in this simulation ${Q}_{{\rm{net}}}$ does not change, Fig. 3b). This clearly suggests that solar insolation is not the only forcing, which impacts the monsoon rainfall. This view is consistent with a previous assertion that both CO${}_{2}$ and orbital changes have influenced climate during the past 150,000 years^50^. In both ORB and GHG, the CWV over India is proportional to the SST over the Arabian Sea (Fig. 3c). Note that for a given SST over the Arabian Sea, CWV is higher in ORB than GHG. This is due to the meridional shift of the ITCZ in ORB, which results from a change in the interhemispheric temperature gradient (see Supplementary Fig. 6). Changes in precession lead to a differential warming of the two hemispheres, whereas changes in GHG lead to a similar warming in both the hemispheres. Similar to GHG, the evolution of $P-E$ in the ICE simulation is due to CWV (Supplementary Fig. 8). This result agrees well with that of a previous study^22^. Thus, we underscore the utility of the diagnostic model in determining the mechanism through which a forcing can affect monsoons.

**Fig. 4:**
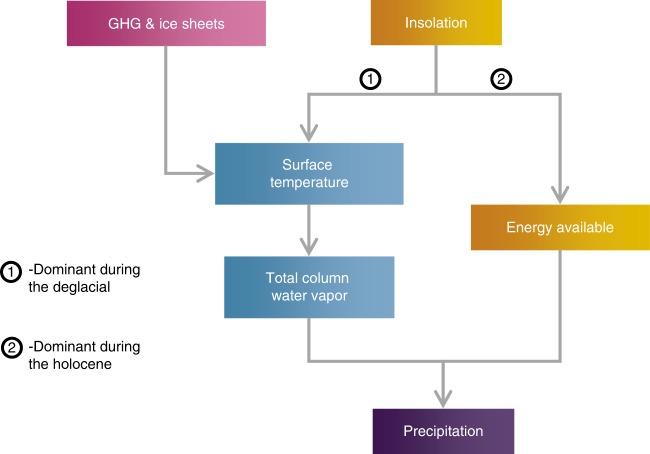
A schematic of the mechanism. The flowchart depicting the mechanism for the Indian monsoon, unraveled by the diagnostic method employed in this study. Insolation influences monsoon by altering both the surface temperature and the local energy available in a column of the atmosphere. The former pathway is dominant during the deglacial period and the latter during the Holocene. The net energy also captures contributions from changes in clouds and therefore represents cloud radiative feedbacks. An indirect effect resulting from changes in surface temperature (TS) amplifies/dampens the solar forcing. Greenhouse gases and ice sheets modulate CWV by affecting the TS.

To summarize, of the three major forcings (orbital, greenhouse gases, and ice sheets), only orbital forcing modulates the net downward energy available over the monsoon region, whereas greenhouse gases and ice sheets affect monsoons through changes in GMS alone. Orbital forcing also influences GMS, but only during colder periods (e.g., LGM and deglacial). We have shown that GMS is a function of total column water vapor. The mechanism is outlined in Fig. 4.

## Discussion

The variability of the Indian summer monsoon occurs on a wide range of timescales from hourly to orbital. The instrumental record exists, however, over the last 150 years. Thus, the variability of monsoon on the centennial scale has been explored mainly with monsoon proxies and time-slice experiments using climate models. The transient simulation—TraCE-21k is a tool to study the evolution of monsoons on the centennial timescales. Dynamic features such as the low-pressure systems and MJO (Madden–Julian oscillation) are known to affect monsoons on synoptic-to-seasonal timescales^51^. The decadal variability of monsoons has also been attributed to the changes in the mean monsoon flow and are related to decadal variations of SST in the Pacific and Atlantic oceans^52,53^. Different modes of variability affect monsoons through different mechanisms. Our results indicate, however, that it is enough to attribute the variations in centennial monsoon to water vapor and energy available. These parameters encapsulate the dynamic and thermodynamic effects on monsoon on these timescales, and thus present a simplified first-order diagnostics.

We have identified quantitatively for the first time the role of feedbacks on the Indian summer monsoon over the last 22,000 years. There has been a long-standing debate between the role of solar forcing and feedbacks through the southern hemisphere in the form of moisture transport^1,5^. Our results highlight that water vapor played a crucial role during the deglacial, but not in the Holocene. The water vapor originated mainly from the Arabian sea and influenced the Indian monsoon through its impact on the GMS. This is the indirect effect of solar forcing on monsoon. The cloud radiative feedbacks were dominant during warmer periods (e.g., the Holocene). Variations in greenhouse gases and ice sheets enhance the indirect effect. Hence, the Indian summer monsoon is more sensitive to insolation during the deglacial than it is during the Holocene. Multiple forcings under future climate change (CO${}_{2}$, aerosols, and deforestation) can influence monsoons. The diagnostic method used in this study can be employed to deduce a mechanism.

## Methods

### Climate model and simulation

The Transient Climate Evolution since the last glacial maximum (21 ka) (TraCE-21K)^31^ is a global climate model simulation using the fully coupled model CCSM3^54^. Transient realistic variations in Earth’s orbit (precession, tilt, and eccentricity), greenhouse gases, ice sheets, and meltwater fluxes were specified. The model has a horizontal resolution of 3.75${}^{\circ }$ (T31) and 26 vertical levels. This model has been shown to reproduce the key features of climate^30,55^ over the last 22,000 years. The long- term trends in the African and East Asian monsoons are also simulated quite well^27,40^. The expansion and intensity of the African monsoon in TraCE-21k are, however, underestimated^39^. There is a dry bias in ISMR in the present-day climate. The bias is −16% with respect to GPCP (Global Precipitation Climatology Project), and −21% with respect to the daily gridded (1${}^{\circ }$ × 1${}^{\circ }$) rainfall dataset of the Indian Meteorological Department (IMD)^56^. Jun–Jul–Aug climatology over the period 1979–2018 and 1951–2013 was used from the GPCP and IMD datasets, respectively. This bias increases to about −33% when Northeast India is included in the study region, without affecting any of our results. Hence, we use the region shown in Fig. 1b. Simulations with variations in only one of these forcings (ORB—orbital only, GHG—greenhouse gas only, ICE—ice sheet only, and MWF—meltwater flux) were also carried out. For this study, we have divided the entire 22,000 years of simulation into 220 continuous but nonoverlapping centuries. Our analysis was performed on this centennially averaged data.

### Calculation of ${\delta }^{18}$O-sw

The ${\delta }^{18}$O obtained from G. Ruber in the KL-126 sediment core^38^ needs to be corrected for influences from SST and ice volume. An independent estimate of SST from alkenones taken from the same sediment core is available^38^. We have taken this SST and interpolated it onto the timestep of ${\delta }^{18}$O. The effect of SST was removed by using the following calibration^57^ for G. Ruber:

3 $${\delta }^{18}{O}_{{\rm{sw}}* }=({T}_{{\rm{Mg}}/{\rm{Ca}}}-14.2)/4.44+{\delta }^{18}O$$

${\delta }^{18}{O}_{{\rm{sw}}* }$ is the SST-corrected ${\delta }^{18}$O. This is then converted to Vienna Standard Mean Ocean Water (VSMOW) by adding $0.2{7}^{{\rm{o}}}{/}_{{\rm{oo}}}$^58^. To correct for ice volume, we have used the following equation^59^:

4 $${\delta }^{18}{O}_{{\rm{sw}}}={\delta }^{18}{O}_{{\rm{sw}}* }+{\mathrm{SL}}* 0.0083$$

where SL is sea level in meters as obtained from Lambeck et al.^60^.

### Diagnostic methdology

The conservation of vertically integrated moisture and moist static energy (MSE) can be used to understand the dynamics of monsoons. The resulting diagnostic model renders precipitation as a function of energy fluxes and vertical stability of the atmosphere^42,43^. The equations for conservation of MSE and moisture for a steady state are as follows:

5 $$\left\langle \nabla \cdot m{\bf{U}}\right\rangle +\left\langle \frac{\partial m\omega }{\partial p}\right\rangle ={Q}_{{\rm{net}}}+{F}_{{\rm{sfc}}}$$

6 $$\left\langle \nabla \cdot q{\bf{U}}\right\rangle +\left\langle \frac{\partial q\omega }{\partial p}\right\rangle =E-P$$

7 $$\left\langle A\right\rangle =- \int_{{P}_{b}}^{P_{t}} A\frac{dp}{g}$$

where the angular brackets ($\left\langle .\right\rangle$) represent vertical integrals. A description of all the other variables is given in Table 1. During the monsoon season, clouds contribute the most to $\alpha$ (reflected shortwave radiation at the top of the atmosphere) and OLR (outgoing longwave radiation). ${Q}_{{\rm{net}}}$ can therefore be expressed as a product of solar insolation $S$ and cloud radiative feedback ($f_{\mathrm{cld}}$) (see Supplementary Fig. 4):

8 $${Q}_{{\rm{net}}}=S(1-\alpha)-{\mathrm{OLR}}=S\left[(1-\alpha)-\frac{{\mathrm{OLR}}}{S}\right]=S\cdot {f}_{{\mathrm{cld}}}$$

Due to its low thermal heat capacity, the storage of heat over land is negligible on longer timescales (about a month). Hence, ${F}_{{\rm{sfc}}}$ is small enough to be neglected (see Supplementary Figs. 11 and 12). Taking the ratio of Eqs. (5) and (6), after multiplying the equation for moisture conservation with the latent heat of vaporization, *L*${}_{{\rm{v}}}$ gives

9 $$P-E=\frac{{Q}_{{\rm{net}}}}{-\frac{\left\langle \nabla \cdot m{\bf{U}}\right\rangle +\left\langle \frac{\partial m\omega }{\partial p}\right\rangle }{{L}_{{\rm{v}}}\left\langle \nabla \cdot q{\bf{U}}\right\rangle +{L}_{{\rm{v}}}\left\langle \frac{\partial q\omega }{\partial p}\right\rangle }}=\frac{{Q}_{{\rm{net}}}}{{\mathrm{GMS}}}$$

10 $${\mathrm{GMS}}=-\frac{\left\langle \nabla \cdot m{\bf{U}}\right\rangle +\left\langle \frac{\partial m\omega }{\partial p}\right\rangle }{{L}_{{\rm{v}}}\left\langle \nabla \cdot q{\bf{U}}\right\rangle +{L}_{{\rm{v}}}\left\langle \frac{\partial q\omega }{\partial p}\right\rangle }=\frac{{Q}_{{\rm{net}}}}{P-E}$$

GMS controls the moisture convergence for a given net downward radiative flux at the top of the atmosphere. The form of the simple diagnostics is similar to that derived by earlier studies^42,43^. The definition of GMS here is, however, different. We have used the conservation of MSE, with all the advection terms included. As depicted in Fig. 2a and Supplementary Fig. 5, we find that GMS is an inverse function of total column water vapor on centennial timescales. Previous studies have derived a similar relation theoretically^43^ for seasonal timescales. The RMSE (root mean square error) in GMS is 0.07, and this translates to an RMSE of 0.19 mm day^−1^ in *P* − *E*. Rainfall ‘*P*’ can therefore be written as follows:

11 $$P=\frac{{Q}_{{\rm{net}}}}{ {\,}^{80.43}/{\mathrm{CWV}}-1.3}+E$$

**Table 1:** Definition of variables.

Variable	Description
$m$	Moist static energy (J kg${}^{-1}$), which is the sum of internal energy, potential energy, and moist energy (${C}_{{\rm{p}}}T+gZ+{L}_{{\rm{v}}}q$)
$\omega$	Vertical component of velocity (Pa s${}^{-1}$)
${\bf{U}}$	Horizontal velocity ($u{\bf{i}}+v{\bf{j}}$) (m s${}^{-1}$)
${Q}_{{\rm{net}}}$	Net downward radiative flux at the top of the atmosphere (in mm day${}^{-1}$; taking the latent heat of vaporization of water as 2.501$\, \times$10${}^{6}$ J kg${}^{-1}$ we get 1 mm day${}^{-1}$ = 28.95 W m${}^{-2}$). It is given by ${{Q}_{{\rm{net}}}=\; S(1\; -\; \alpha)\; -\; {\mathrm{OLR}}}$, where $S$ is insolation, $\alpha$ is the shortwave reflectivity at the top of the atmosphere, and OLR is the outgoing longwave radiation
${F}_{{\rm{sfc}}}$	Net surface energy fluxes into the atmosphere (mm day${}^{-1}$). It is the sum of surface radiative, latent, and sensible heat fluxes
$q$	Specific humidity (kg kg${}^{-1}$)
$P$	Precipitation rate (mm day${}^{-1}$)
$E$	Evaporation rate (mm day${}^{-1}$)
${P}_{b}$	Pressure at the bottom of the atmospheric column (Pa)
${P}_{{\rm{t}}}$	Pressure at the top of the atmospheric column (Pa)
$g$	Acceleration due to gravity (m s${}^{-2}$)

This table describes all the variables used in the Methods section

### Relative contribution of water vapor and cloud feedback

The ratio of Eq. (2) written for a given climate is taken with that for a reference climate. Subscript (0) here represents the reference climate (we have taken preindustrial climate as a reference)

12 $$\frac{P-E}{{(P-E)}_{0}}=\frac{S}{{S}_{0}}\cdot \frac{{f}_{{\mathrm{cld}}}}{{f}_{{\mathrm{cld0}}}}\cdot \frac{{f}_{{\rm{cwv}}}}{{f}_{{\rm{cwv0}}}}$$

Since this is a ratio, the departure of each term in the above equation from unity, is the fractional change in the respective parameter, i.e.,

13 $${1\; -\frac{a}{{a}_{0}}=\frac{{a}_{0}-\; a}{{a}_{0}}=\frac{\Delta a}{{a}_{0}}}$$

where $a$ is any of the parameters of the diagnostic model: $P-E$, $S$, *f*_cld_, or *f*_cwv_. This departure is, therefore, used to quantitatively identify whether cloud feedback or indirect effect of water vapor is dominant.

### Calculation of the relative contribution of GMS and energy

The following equation, which was derived earlier^16^, is used to evaluate the relative role of GMS and ${Q}_{{\rm{net}}}$ in determining the changes in monsoon between two climate states:

14 $$\underbrace{\frac{\Delta (P-E)}{P-E}}_{{\mathrm{Change}}\; {\rm{in}}\; P-E}\, = \underbrace{\frac{\frac{\Delta Q}{Q}}{1+\frac{\Delta G}{G}}}_{{\mathrm{Contribution}}\;{\rm{from}}\,{Q}_{net}}+ \underbrace{\frac{-\frac{\Delta G}{G}}{1+\frac{\Delta G}{G}}}_{{\mathrm{Contribution}}\;{\rm{from}}\,{\rm{GMS}}}$$

$Q$ and $G$ are short forms used in place of ${Q}_{{\rm{net}}}$ and GMS, respectively. $\Delta$ represents the difference of a quantity between its value at a period in question and a reference climate (we have taken preindustrial climate (1750 A.D. to 1850 A.D.) as a reference). The term on the LHS is the fractional change in $P-E$. The first term on the RHS gives the contribution from energy, whereas the second term gives the contribution from GMS. For the GHG and ICE simulations, since insolation does not change, ${Q}_{{\rm{net}}}$ remains a constant (Fig. 3b and Supplementary Fig. 8). All of the variations in $P-E$, therefore, are related to GMS. Since GMS for these simulations is a function of CWV only (see Supplementary Fig. 9), we can attribute the changes in monsoon to CWV. In the ORB simulation, both ${Q}_{{\rm{net}}}$ and GMS contribute to changes in moisture convergence. The role of GMS is, however, dominant. GMS was evaluated based on the fit for ORB (not shown).

## Supplementary information

Supplementary Information

## Data Availability

The ${\delta }^{18}$O data used in Fig. 1a are obtained from 10.1594/PANGAEA.735053. The TraCE-21k dataset, analyzed in this study, can be downloaded from the Earth System Grid (National Center for Atmospheric Research) (https://www.earthsystemgrid.org/project/trace.html). The GPCP precipitation data used in this study were provided by the NOAA/OAR/ESRL PSD, Boulder, Colorado, USA, from their website at https://www.esrl.noaa.gov/psd/. The IMD rainfall dataset was obtained from http://www.imd.gov. The data from LOVECLIM DG_ns simulation are available on apdrc (http://apdrc.soest.hawaii.edu/datadoc/dg_ns.php). The CMIP5/PMIP3 datasets are publicly available at https://esgf-data.dkrz.de/search/cmip5-dkrz/.

## Code availability

The codes are available from the corresponding author C.J. on request.
