# Supplementary material for: Modulation of Indian monsoon by water vapor and cloud feedback over the past 22,000 years
Source: Nat Commun. 2019 Dec 13;10:5701. doi: 10.1038/s41467-019-13754-6 (PMC6911089; doi:10.1038/s41467-019-13754-6)
Supplement: Supplementary file 1 — Supplementary Information [file 41467_2019_13754_MOESM1_ESM.pdf]

Supplementary information

Modulation of the Indian monsoon by water vapor and cloud feedback over the past 22,000 years

Jalihal et al.

Supplementary Note 1: Climate evolution over the last 22,000 years.

The present ice age is characterized by periodic shifts into cold and warm phases known as the glacial and the interglacial periods, respectively. During the glacial periods, GHG concentrations drop, and ice sheets expand, whereas, during the interglacial periods, GHG concentrations rise and ice sheets retreat [1,2]. From around 110,000 years to about 15,000 years ago, Earth was in a glacial period [3] with the peak occurring at about 21,000 years ago [4–6] known as the Last Glacial Maximum (LGM). The last 21,000 years can be divided into 2 periods: the deglacial (21 ka –11 ka) [7] and the Holocene (11 ka –present) [8].

Proxy–based reconstructions suggest that the deglacial period is characterized by the receding of ice sheets and increasing greenhouse gases [7]. The melting of ice sheets released freshwater into the oceans resulting in global sea-level rise. By the end of the deglacial, most of the ice sheets over Europe and North America had decayed [5,6]. The freshwater released into the Atlantic led to a slowdown of the Atlantic Meridional Overturning Circulation (AMOC) [9,10] and was responsible for the large centennial to millennial–scale climate fluctuations during this period [11–15]. CO_2 increased from about 180 ppm to 260 ppm [16]. The summer insolation intensified and attained its highest value towards the end of the deglacial, due to the precession of the Earth's axis. The Holocene period has been relatively stable. The variations in the ice sheet extent and greenhouse gases were quite small. CO_2 increased gradually by about 20 ppm [16], and the northern hemisphere summer insolation declined during this period.

Supplementary Figures

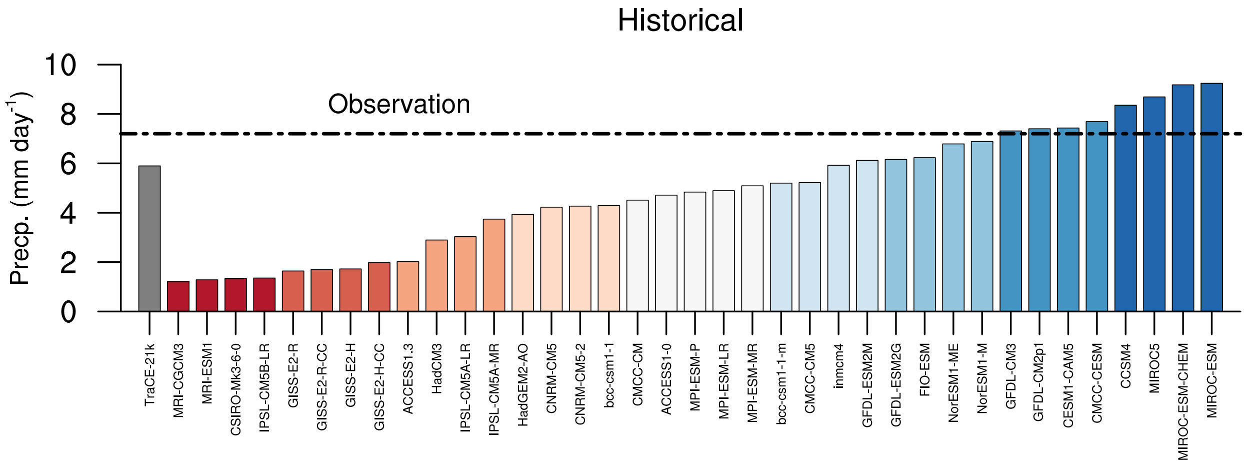

Supplementary Figure 1: TraCE-21k vs CMIP5. The bar chart shows a comparison between the Jun-Jul-Aug (JJA) mean precipitation over the domain (10°–29°N and 70°–85°E; land only points) (see main Fig. 1b for the domain chosen) for all the models that participated in the CMIP5 historical experiment. JJA mean precipitation over the same domain from the GPCP dataset is shown for reference.

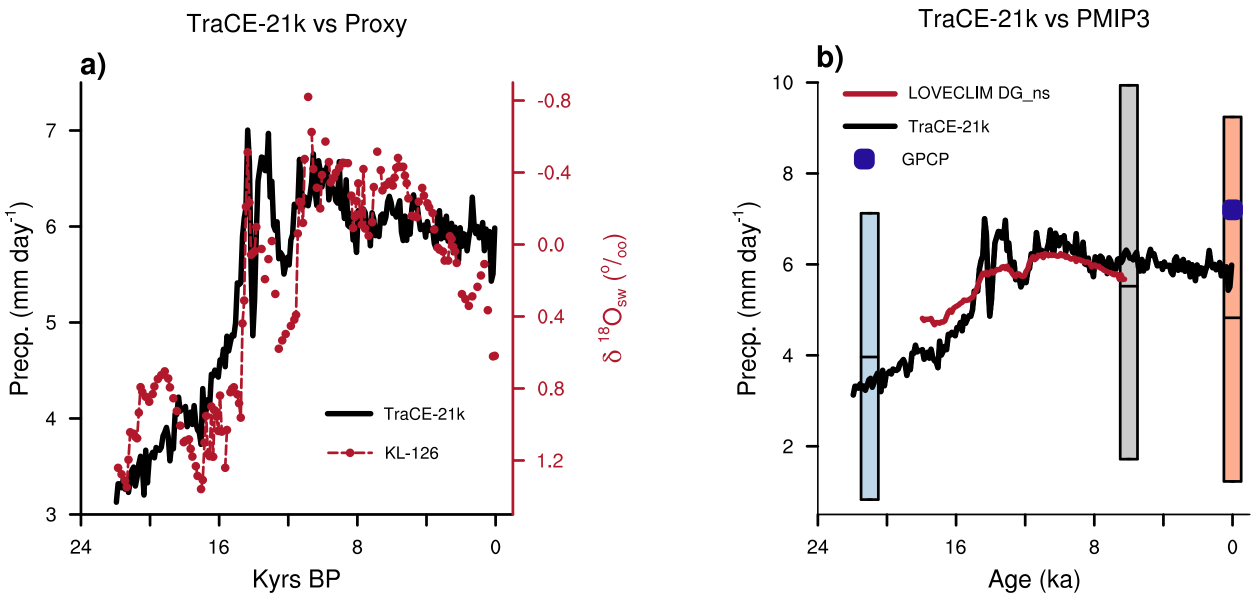

Supplementary Figure 2: Model comparison with observation and CMIP5. The timeseries of Jun-Jul-Aug (JJA) mean precipitation over India (10°–29°N and 70°–85°E; land only points) in black, shown along with **a,** $\delta\ ^{18}O_{sw}$ from the sediment core KL-126 in the North Bay of Bengal [17] **b,** JJA mean precipitation over India from LOVECLIM DG_ns [18] simulation in red. The boxes indicate the spread in the PMIP3/CMIP5 models. The extremes of the boxes correspond to the models with the highest and lowest precipitation, whereas the black line inside the box indicates the multimodel mean. The blue, black, and red boxes are based on the PMIP3–LGM, PMIP3–MH and CMIP5 historical simulations.

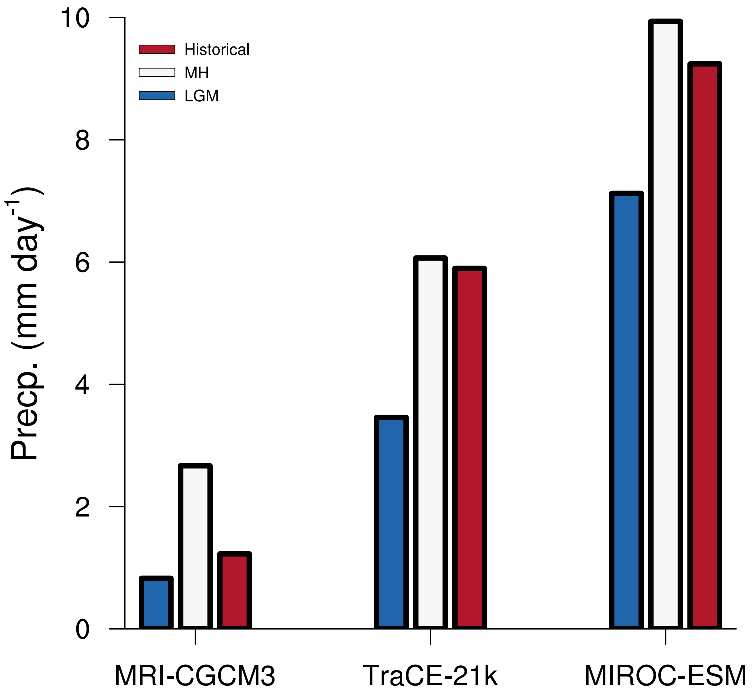

Supplementary Figure 3: TraCE-21k vs a model with dry and wet bias. The bar chart shows a comparison of the Jun-Jul-Aug (JJA) mean precipitation at LGM, MH and the historical period in blue, white and red bars respectively over the domain (10°–29°N and 70°–85°E; land only points). We have taken a model with the largest dry bias (MRI-CGCM3), and another model with the largest wet bias (MIROC-ESM) in their historical simulations.

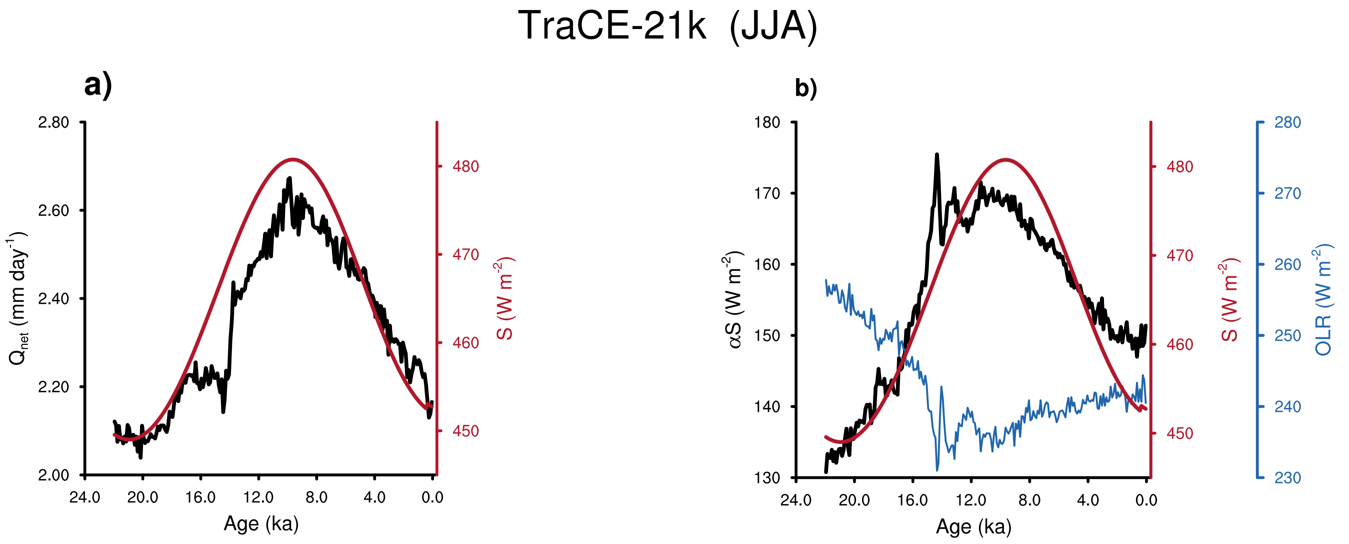

Supplementary Figure 4: Relation between net energy and insolation. The time series of Jun-Jul-Aug (JJA) mean (**a**) net downward energy at the top of the atmosphere (Q_{net}) in black and insolation (S) in red, (**b**) reflected short wave at the top of atmosphere (αS) in black, S in red, and outgoing longwave radiation (OLR) in blue. These parameters are averages over India (10°-29°N; 70°-85°E; land only points) from the TraCE-21k.

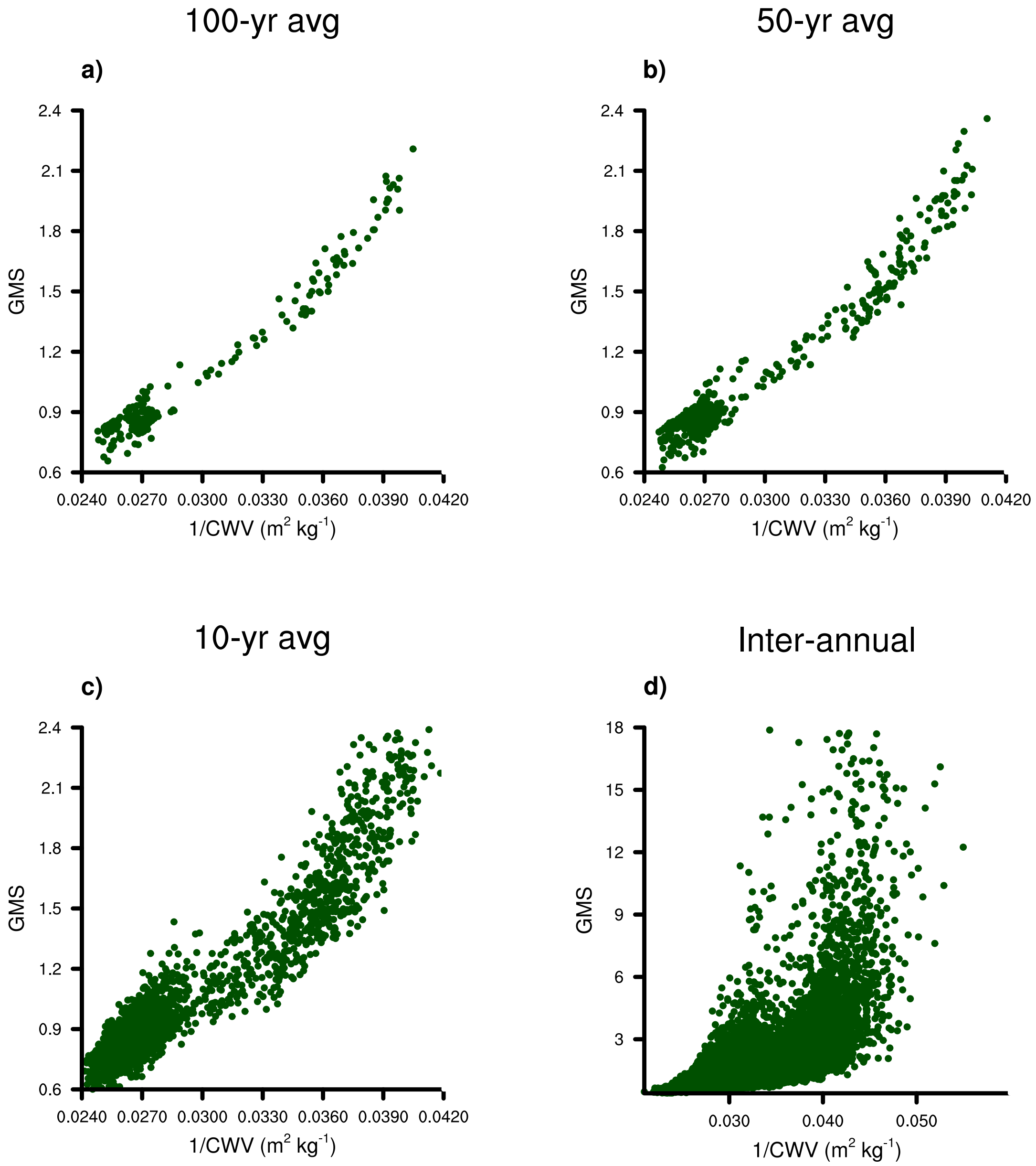

Supplementary Figure 5: GMS as a function of water vapor. The relation between Jun-Jul-Aug (JJA) mean gross moist stability (GMS) and total column water vapor (CWV) over India for (**a**) 100-year averages, (**b**) 50-year averages, (**c**)10-year averages, and (**d**) seasonal average for every year.

Supplementary Table 1: Relative contribution of solar forcing and the feedbacks. Based on the analysis detailed in the methods section, Eq. 12 (from the main manuscript) is used to quantify the relative role of solar forcing (S) cloud feedback (f_{cld}), and the indirect effect of water vapor (f_{cwv}) on moisture convergence ($P - E$) over India. Subscript 0 represents the reference climate. Climatology is based on the 1000–year average (Jun-Jul-Aug), starting from the specified year (eg. 10 ka implies climatology over 10 ka to 9 ka). All values are evaluated over the box depicted in Fig. 1b of the main manuscript. The departure of the ratio from unity is the fractional change of the parameter between the reference climate and the climate of interest. This is used to identify the relative dominance of cloud feedback or indirect effect of water vapor. The dominant parameter is shown in bold font.

	$\frac{\mathbf{P-E}}{\mathbf{P-E_0}}$	$\frac{\mathbf{S}}{\mathbf{S_0}}$	$\frac{\mathbf{f}_{\text{cld}}}{\mathbf{f}_{\text{cld0}}}$	$\frac{\mathbf{f}_{\text{cwv}}}{\mathbf{f}_{\text{cwv0}}}$
$\frac{\mathbf{1ka}}{\mathbf{10ka}}$	0.84	0.95	**0.91**	0.97
$\frac{\mathbf{10ka}}{\mathbf{22ka}}$	3.2	1.07	1.16	**2.6**

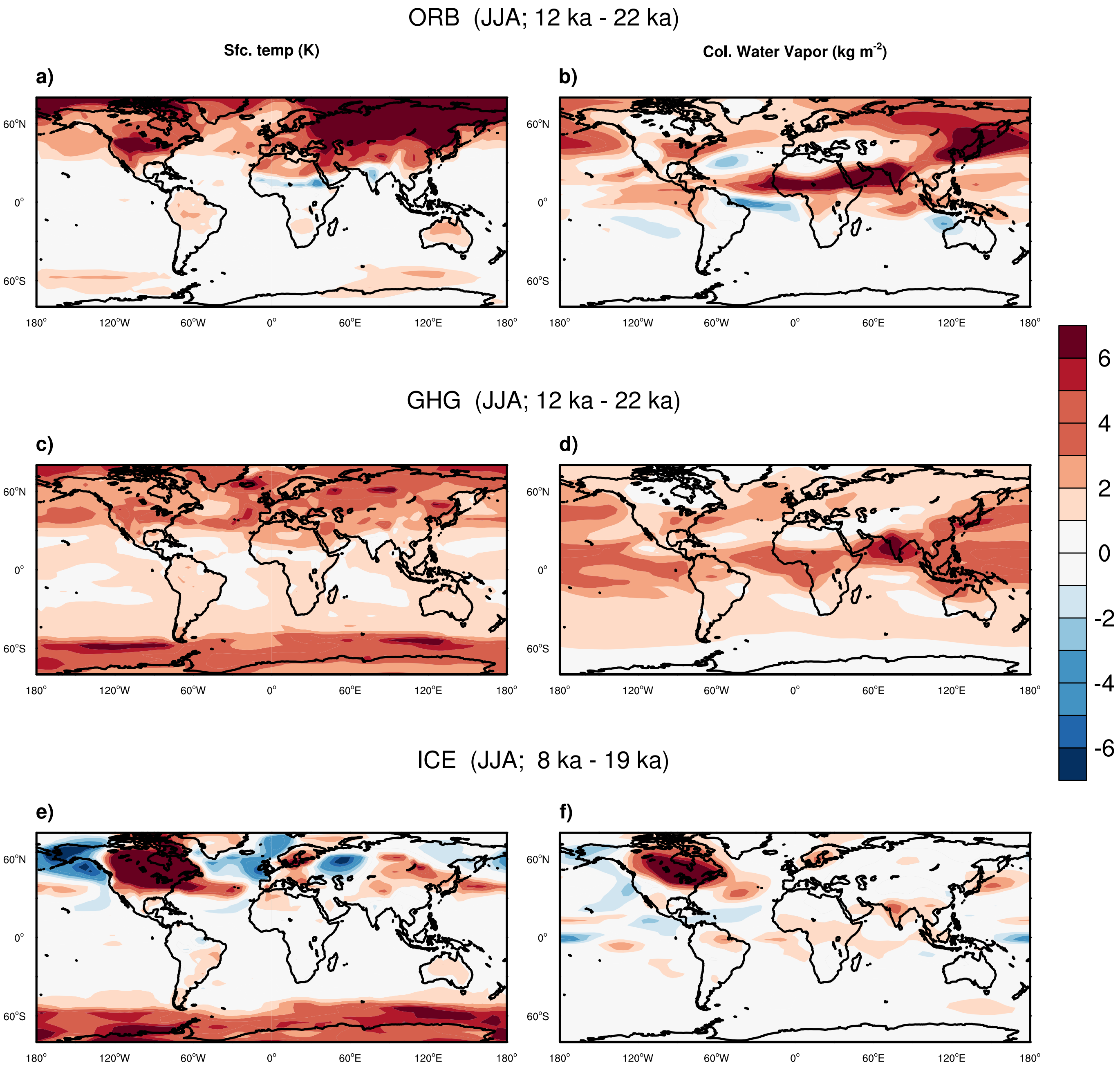
ORB (JJA; 12 ka - 22 ka)
Sfc. temp (K)
Col. Water Vapor (kg m^{-2})
a)
b)
GHG (JJA; 12 ka - 22 ka)
c)
d)
ICE (JJA; 8 ka - 19 ka)
e)
f)
60°N
0°
60°S
180°
120°W
60°W
0°
60°E
120°E
180°
6
4
2
0
-2
-4
-6

Supplementary Figure 6: Response of surface temperature and moisture. The difference between the Jun-Jul-Aug (JJA) climatology at 10 ka (period: 10 ka–9 ka) and 1 ka (period: 1 ka–0 ka) for the simulations with individual forcings. The plots (**a, c**, and **e**) depict differences in surface temperature and the plots (**b, d**, and **f**) show differences in total column water vapor. (**a**) and (**b**) are for the ORB (orbital forcing only) simulation, (**c**) and (**d**) depict the GHG (greenhouse gas forcing only) simulation, (**e**) and (**f**) show the ICE (ice sheet forcing only) simulation.

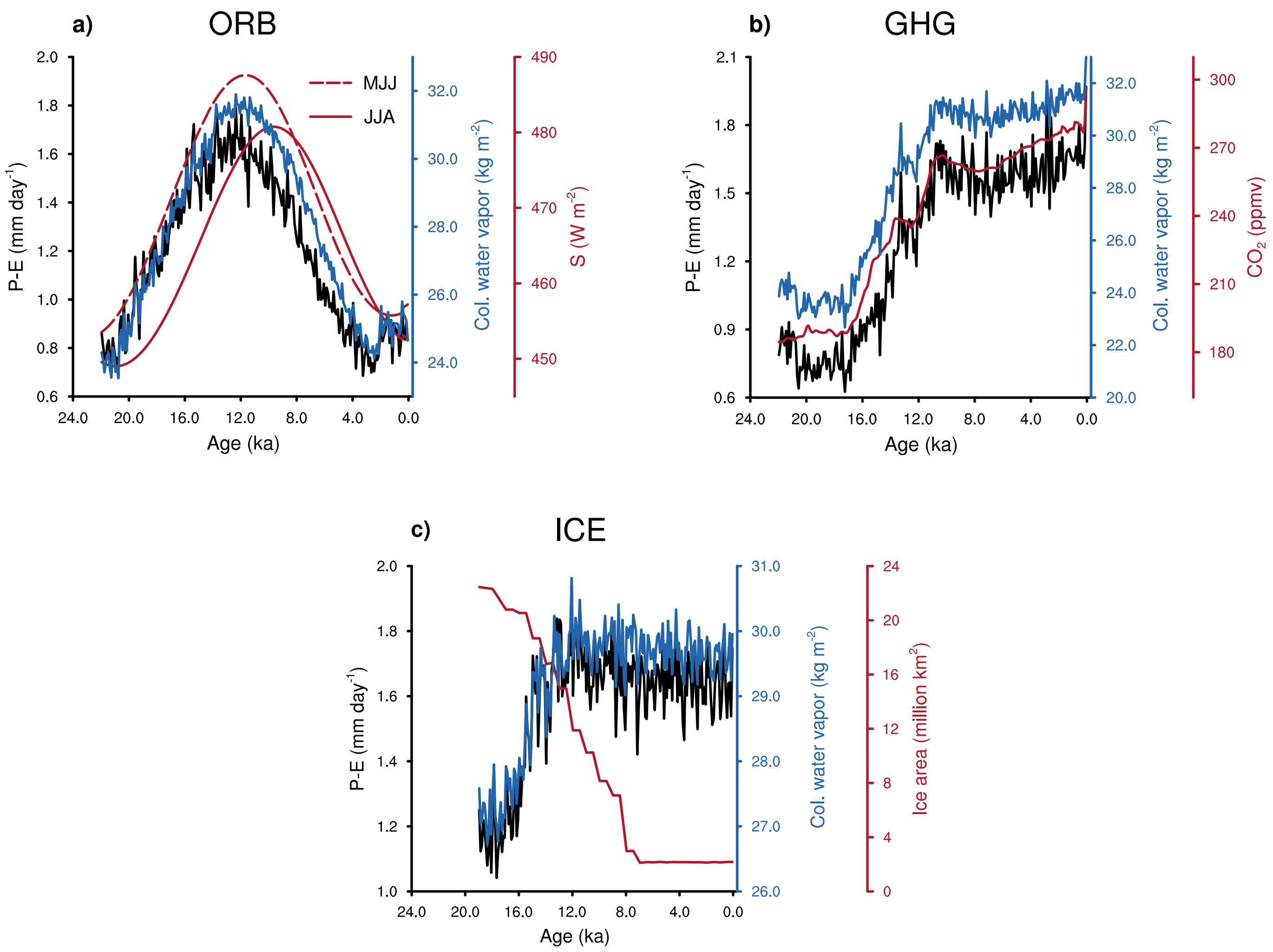

Supplementary Figure 7: Variations in individual forcings and moisture convergence. The time series of total column water vapor (CWV) in blue, moisture convergence ($P - E$) in black, and forcing in red over India, for the (**a**) ORB (orbital only) simulation, (**b**) GHG (greenhouse gas only) simulation, and (**c**) ICE (ice sheet only) simulation. The area covered by ice is evaluated over the domain (40°–90°N and 0°–30°E). In (**a**) it is shown that the total column water vapor corresponds to May–Jun–Jul (MJJ) insolation (dashed red line) rather than to the Jun–Jul–Aug (JJA) insolation (thick red line). All of the parameters are estimated for the mean over JJA.

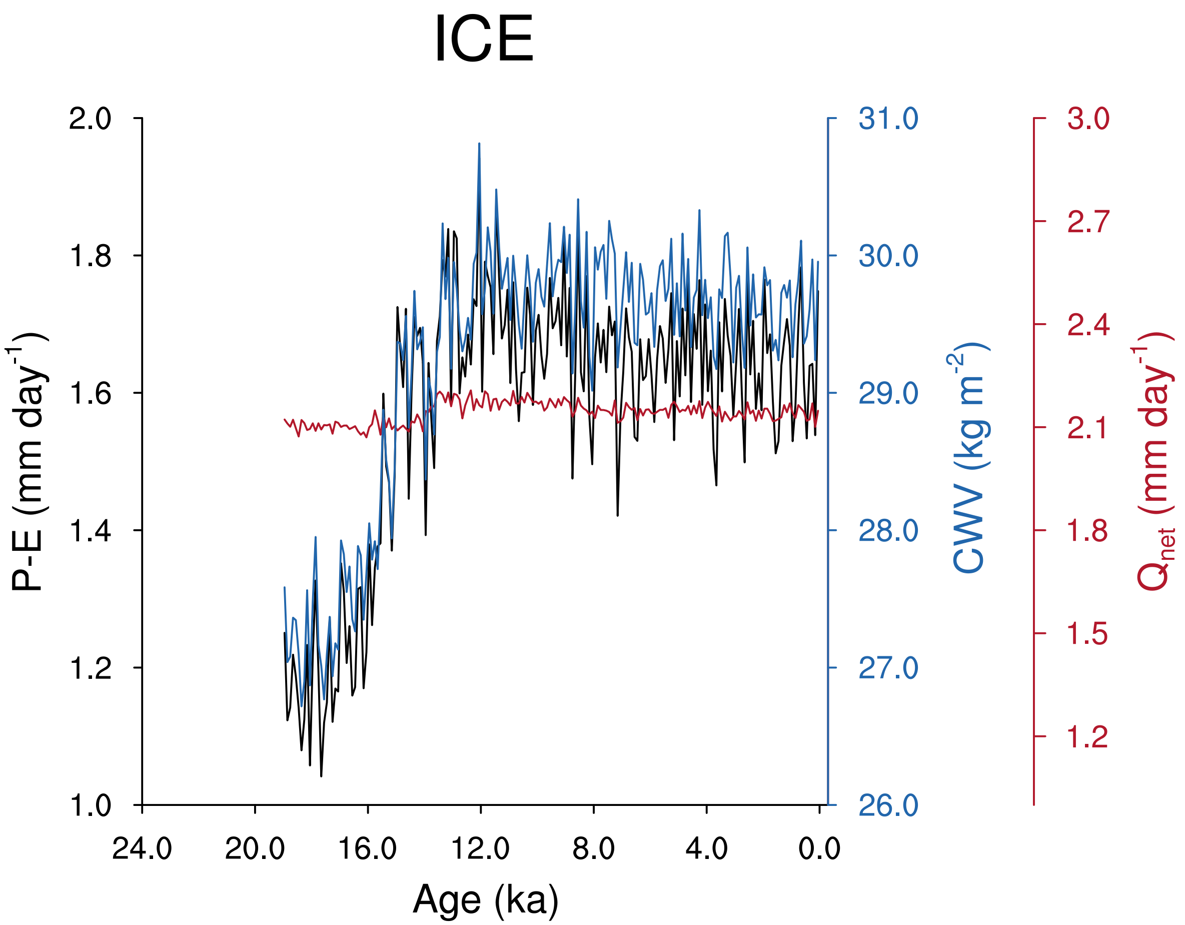

Supplementary Figure 8: Role of net energy and water vapor in ICE simulation. The time series of moisture convergence ($P - E$) in black, total column water vapor (CWV) in blue, net downward radiative fluxes at the top of the atmosphere (Q_{net}) in red. All of these parameters are evaluated over India, for the average of Jun-Jul-Aug. This figure is for the ICE (ice sheet only forcing) simulation.

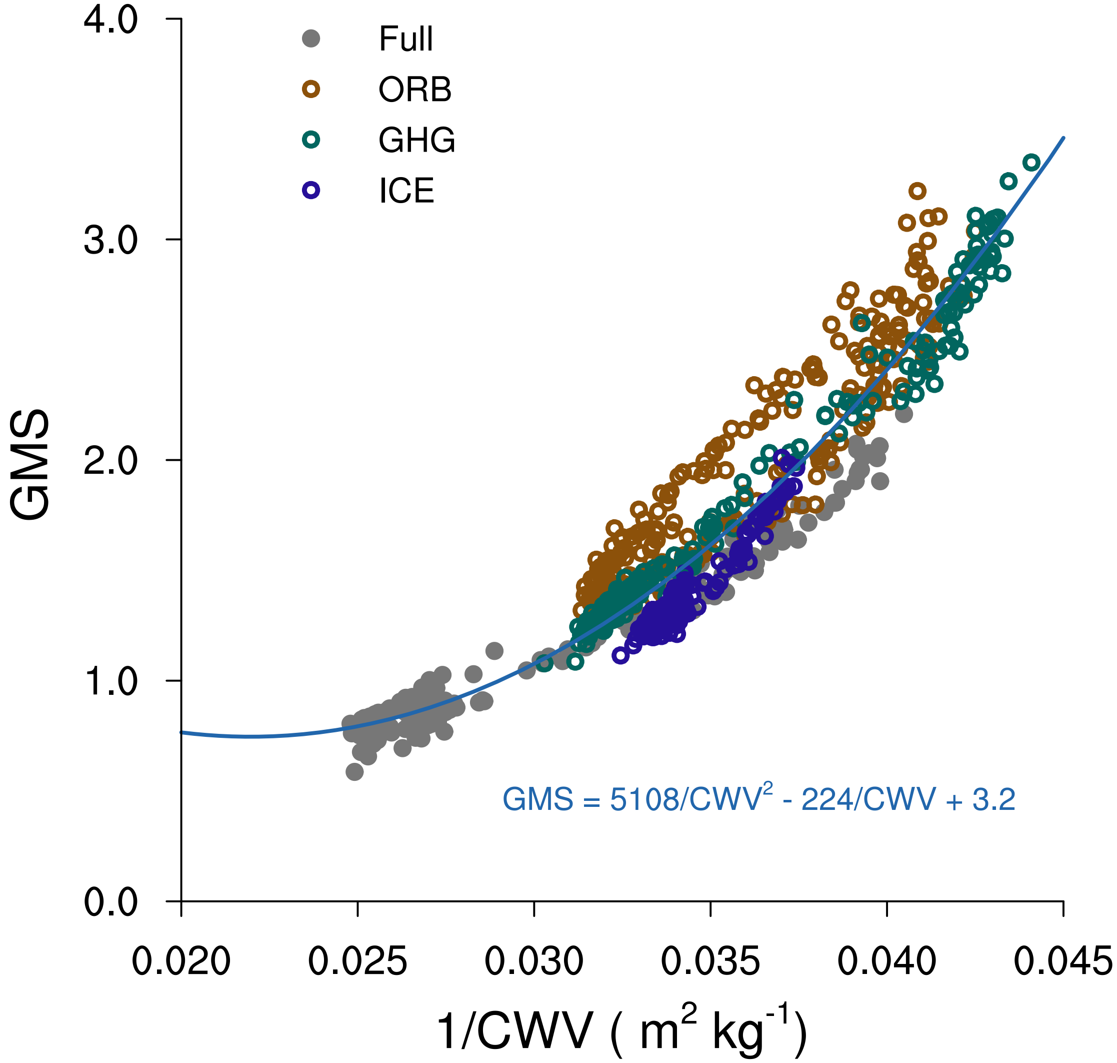

Supplementary Figure 9: Relation between GMS and water vapor for all simulations. The scatter of gross moist stability (GMS) with total column water vapor (CWV) over India. Grey filled circles are for the TraCE-21k simulation. The brown, green, and violet open circles denote ORB, GHG, and ICE simulations, respectively.

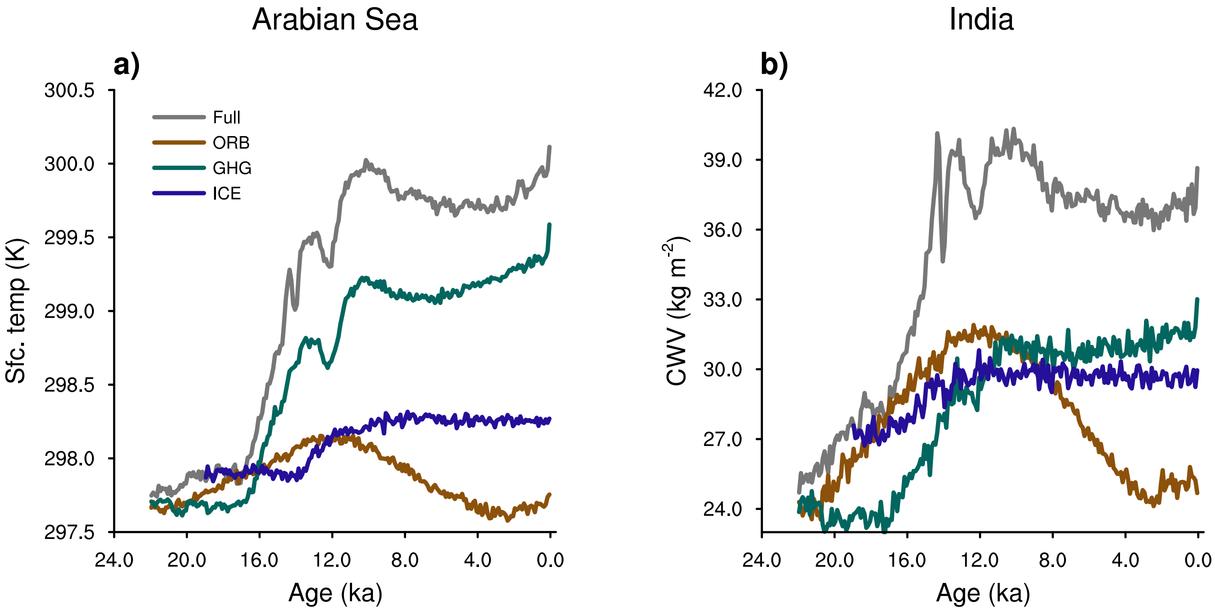

Supplementary Figure 10: Contribution of individual forcings. The Jun-Jul-Aug (JJA) mean time series of **a,** surface temperature over the domain (0°-29°N; 50°-70°E), and **b,** total column water vapor (CWV) over India (10°-29°N; 70°-85°E; land-only grids). The lines in grey, brown, green, and violet correspond to the TraCE-21k, ORB, GHG, and ICE simulations. The ORB, GHG, and ICE simulations are the transient simulations where only one of the forcing (orbital, greenhouse gases, and ice sheets respectively) was varied.

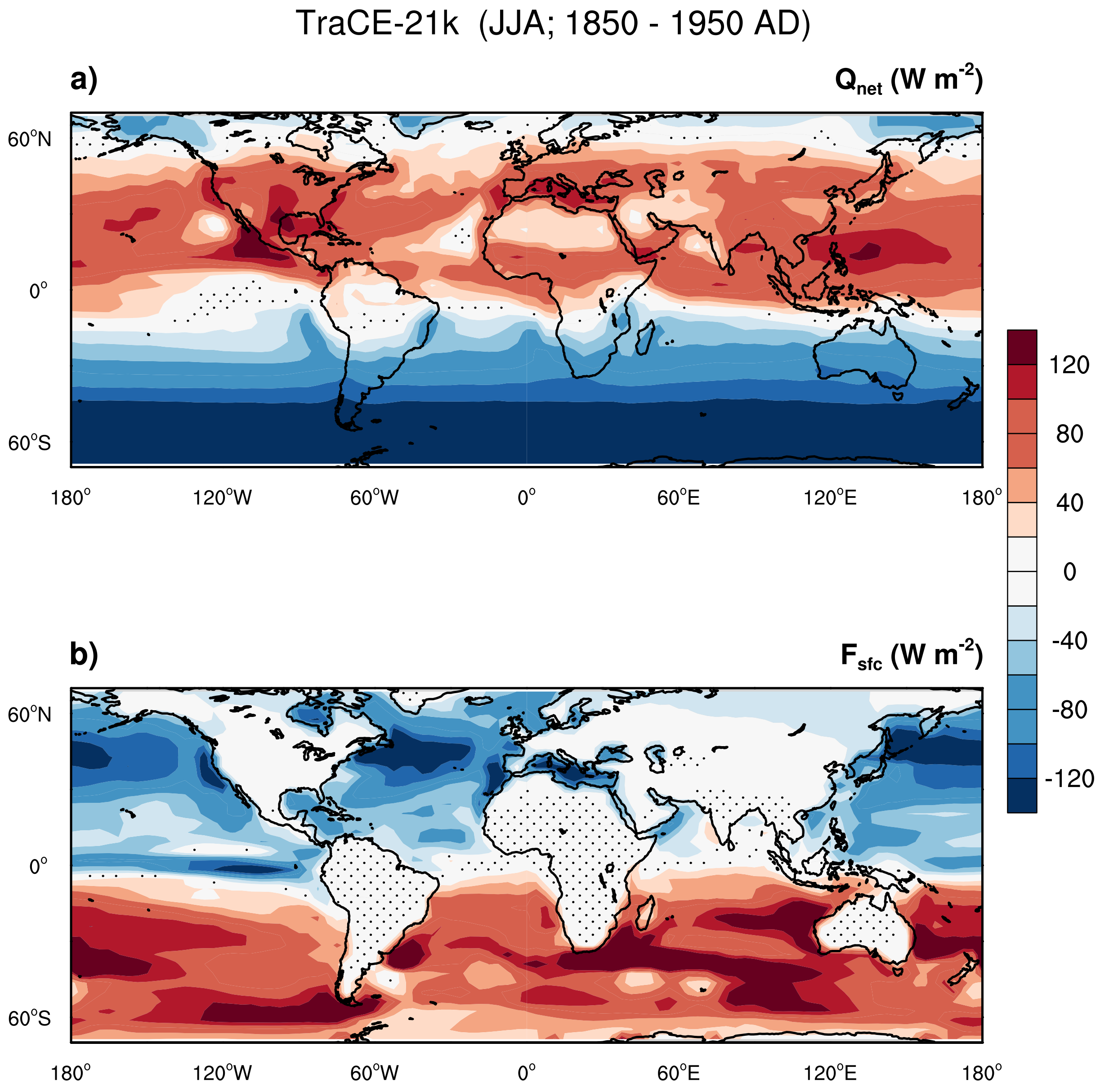

Supplementary Figure 11: Energy fluxes at the surface vs top of the atmosphere. The Spatial plot of the Jun-Jul-Aug (JJA) mean climatology in TraCE–21k calculated over the time period (1850 A.D to 1950 A.D) for (**a,**) net downward energy fluxes at the top of the atmosphere (Q_{net}), and (**b,**) net upward energy fluxes at the surface (F_{sfc}). Positive values represent the addition of energy into the atmosphere. Stippling depicts grids with energy fluxes of small values (between -5 and 5 W m^{-2}).

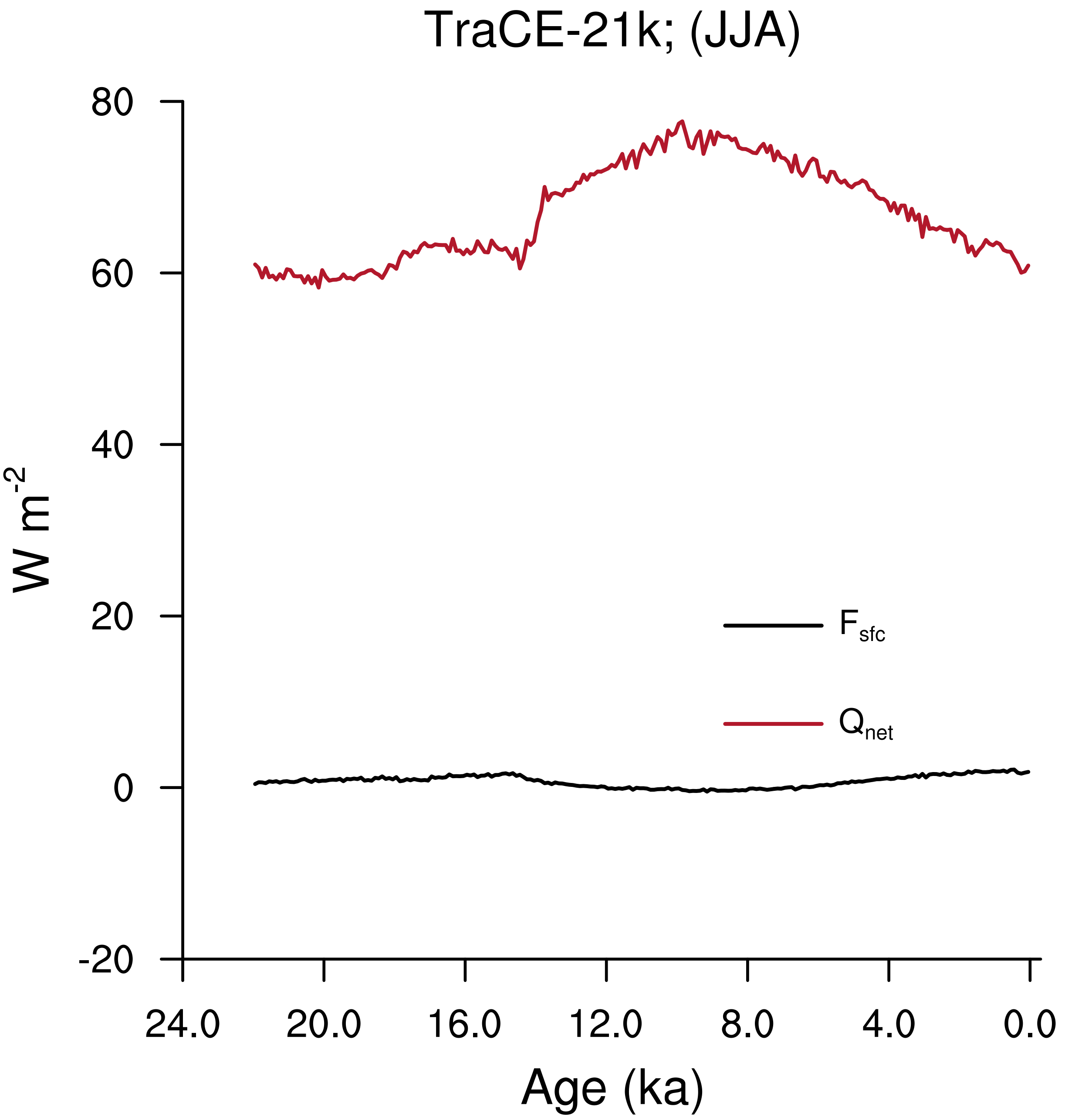

Supplementary Figure 12: Energy fluxes at the top and bottom of the atmosphere. The time series of net downward energy at the top of the atmosphere (Q_{net}) in red and net surface energy fluxes (F_{sfc}) in black averaged over India, and for the months Jun-Jul-Aug (JJA), from the TraCE-21k.

Supplementary Methods: Results from LOVECLIM DG_ns

Another transient simulation LOVECLIM DG_ns [18] (18 ka to 6.2 ka), using a model of intermediate complexity EC-Bilt, agrees with TraCE21k not only in terms of the changes in precipitation over India but also in terms of the mechanism. To find out the relative dominance of Q_{net} and GMS during different periods, we have done the following:

$$P - E = \frac{Q_{\text{net}}}{GMS} \tag{1}$$

The above equation for a reference period (taken to be the most recent century in the simulation .ie. climatology of the period 6.3 ka to 6.2 ka).

$$(P - E)_0 = \frac{Q_{\text{net0}}}{GMS_0} \tag{2}$$

Taking a ratio of the two equations:

$$\frac{P - E}{(P - E)_0} = \frac{Q_{\text{net}}}{Q_{\text{net0}}} * \frac{GMS_0}{GMS} \tag{3}$$

The relative dominance of the first or the second term indicates the dominance of Q_{net} or GMS during a particular period with respect to the reference period. LOVECLIM DG_ns also has a GMS dominant regime (18 ka to 9 ka) and a Q_{net} dominant regime (9 ka to 6 ka). GMS in this simulation is also a linear function of water vapor (since only near-surface specific humidity was

available, we have used it in place of total column water vapor).

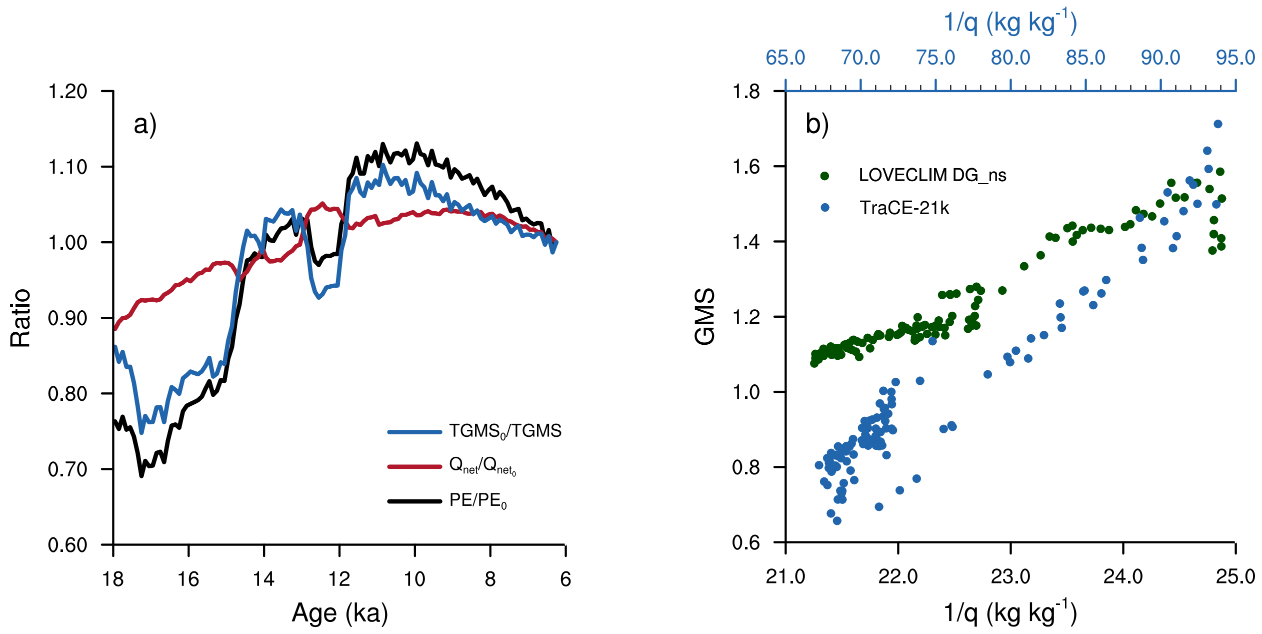

Supplementary Figure 13: Results from LOVECLIM DG_ns. The time series of Jun-Jul-Aug (JJA) mean moisture convergence ($P - E$), net downwared radiative fluxes at the top of the atmosphere (Q_{net}), and gross moist stability (GMS) normalized with their respective values for the most recent climatology in the simulation (period: 6.3 ka to 6.2 ka) is depicted in (**a**). A scatter of JJA mean GMS and $1/q$ (specific humidity at the surface) for LOVECLIM DG_ns in green is shown in (**b**). The relation between GMS and near-surface specific humidity from the TraCE-21k is also shown in blue for comparison. Centennially averaged data over the domain (10°-29°N; 70°-85°E; land-only grids) is used for both the plots.

Supplementary References

1. Lisiecki, L. E. & Raymo, M. E. A pliocene-pleistocene stack of 57 globally distributed benthic δ18o records. *Paleoceanography* **20** (2005).

2. Kawamura, K. *et al.* Northern hemisphere forcing of climatic cycles in antarctica over the past 360,000 years. *Nature* **448**, 912916 (2007).

3. Severinghaus, J. P. & Brook, E. J. Abrupt climate change at the end of the last glacial period inferred from trapped air in polar ice. *Science* **286**, 930–934 (1999).

4. CLIMAP Project Members. LGM CLIMAP sea surface temperature (1976). URL `https://doi.org/10.1594/PANGAEA.64427`.

5. CLIMAP Project Members. Relative abundance of planktic foraminifera in the 120 kyr time slice reconstruction of sediment core GIK12392-1 (1981). URL `https://doi.org/10.1594/PANGAEA.51983`.

6. Peltier, W. Global glacial isostasy and the surface of the ice-age Earth: the ICE-5G (VM2) model and GRACE. *Annu. Rev. Earth Planet. Sci.* **32**, 111–149 (2004).

7. Clark, P. U. *et al.* Global climate evolution during the last deglaciation. *Proceedings of the National Academy of Sciences* **109**, E1134–E1142 (2012).

8. Walker, M. *et al.* Formal definition and dating of the gssp (global stratotype section and point) for the base of the holocene using the greenland ngrip ice core, and selected auxiliary records.

Journal of Quaternary Science: Published for the Quaternary Research Association **24**, 3–17 (2009).

9. Stommel, H. Thermohaline convection with two stable regimes of flow. *Tellus* **13**, 224–230 (1961).

10. Rahmstorf, S. *et al.* Thermohaline circulation hysteresis: A model intercomparison. *Geophysical Research Letters* **32** (2005).

11. Broecker, W. S., Peteet, D. M. & Rind, D. Does the ocean–atmosphere system have more than one stable mode of operation? *Nature* **315**, 2126 (1985).

12. Cheng, H., Sinha, A., Wang, X., Cruz, F. W. & Edwards, R. L. The global paleomonsoon as seen through speleothem records from asia and the americas. *Climate Dynamics* **39**, 1045–1062 (2012).

13. Wang, P. *et al.* The global monsoon across timescales: coherent variability of regional monsoons. *Climate of the Past* **10**, 2007–2052 (2014).

14. Liu, Z. *et al.* Transient simulation of last deglaciation with a new mechanism for Bølling-Allerød warming. *Science* **325**, 310–314 (2009).

15. Otto-Bliesner, B. L. *et al.* Coherent changes of southeastern equatorial and northern African rainfall during the last deglaciation. *Science* **346**, 1223–1227 (2014).

16. Joos, F. & Spahni, R. Rates of change in natural and anthropogenic radiative forcing over the past 20,000 years. *Proceedings of the National Academy of Sciences* **105**, 1425–1430 (2008).

17. Kudrass, H., Hofmann, A., Doose, H., Emeis, K. & Erlenkeuser, H. Modulation and amplification of climatic changes in the Northern Hemisphere by the Indian summer monsoon during the past 80 ky. *Geology* **29**, 63–66 (2001).

18. Menviel, L., Timmermann, A., Timm, O. E. & Mouchet, A. Deconstructing the last glacial termination: the role of millennial and orbital-scale forcings. *Quaternary Science Reviews* **30**, 1155–1172 (2011).
